# Key drivers of reversal of trend in childhood anaemia in India: evidence from Indian demographic and health surveys, 2016–21

**DOI:** 10.1186/s12889-023-16398-w

**Published:** 2023-08-18

**Authors:** S. K. Singh, H. Lhungdim, Chander Shekhar, L. K. Dwivedi, S. Pedgaonkar, K. S. James

**Affiliations:** 1 Department of Survey Research and Data Analytics, International Institute for Population Sciences, Mumbai, India; 2 Department of Public Health & Mortality Studies, International Institute for Population Sciences, Mumbai, India; 3 Department of Fertility Studies, International Institute for Population Sciences, Mumbai, India; 4 Department of Family & Generations, International Institute for Population Sciences, Mumbai, India; 5 International Institute for Population Sciences, Mumbai, India

**Keywords:** Key drivers, Reversal of trends, Childhood anaemia, Under-five children, India

## Abstract

**Aim:**

Recent National Family Health Survey results portray striking improvements in most population and health indicators, including fertility, family planning, maternal and child health, gender treatment, household environments, and health insurance coverage of the Pradhan Mantri Jan Arogya Yojana (PM-JAY), with all India resonance. However, the prevalence of any anaemia (< 11 g/dl) among children under age five has exhibited a reversed trajectory in recent years. Therefore, the present study explores key drivers of the reversal of the trend in the prevalence of childhood anaemia between 2015 and2021.

**Methods:**

Data of four rounds of the National Family Health Survey (NFHS) were used to show the overall trend of anaemia among children. However, for the analysis of key drivers of the reversal trend of childhood anaemia, only the recent two rounds (NFHS-4 & NFHS-5) were used. Descriptive, bivariate multivariable analysis and Fairlie decomposition model were used to explore the drivers of the reversal of the trend in childhood anaemia.

**Results:**

During the past two decades, India has seen a decline in the prevalence of childhood anaemia (NFHS-2 to NFHS-4). However, a reversal of trend was observed recently. The prevalence of anaemia among children aged 6–59 months increased from 59 percent in NFHS-4 to 67 percent in NFHS-5. In addition, the prevalence of mild anaemia increased from 23.3 percent in NFHS-2 to 28.7 percent in NFHS-5. However, the prevalence of moderate and severe anaemia declined considerably from NFHS-2 (40 percent and 4.1 percent) to NFHS-4 (28.7 percent and 1.6 percent), but showed an increase in the prevalence in NFHS-5 (36.3 percent and 2.2 percent). Among others, mothers’ educational attainment, anaemia status and socio-economic status emerge as the key drivers of the change in the prevalence of childhood anaemia.

**Conclusion:**

These findings may have vital implications for the ongoing Anaemia Mukt Bharat Programme, one of the government's dream projects in India.

**Supplementary Information:**

The online version contains supplementary material available at 10.1186/s12889-023-16398-w.

## Introduction

Anaemia is a condition in which the number of red blood cells or the haemoglobin concentration within them is lower than normal, which results in the diminished oxygen-carrying capacity of the blood to meet the needs of body tissues [1]. The usual symptoms of anaemia are fatigue, weakness, dizziness, and shortness of breath. Anaemia is considered a proxy indicator of iron deficiency, which is usually caused by poor iron intake and low iron bioavailability [2]. Anaemia is a significant public health problem that is disproportionately concentrated in developing countries worldwide. In addition, globally, more than 40 percent of under-five children are suffering from anaemia [1, 3, 4].

Anaemia is known to be multifactorial in its etiology, with causes including specific nutritional deficiencies (of iron, folic acid, vitamin B-12, vitamin A, riboflavin, pyridoxine, zinc, copper), infections, hemoglobinopathies, as well as inflammation related to chronic disease [5]. However, similar to other developing countries, anaemia control programmes in India, since their initiation in 1970, have continued to revolve around iron-folic acid (IFA) supplementation because iron deficiency is still considered the major cause of anaemia [6]. However, analyses from the Biomarkers Reflecting Inflammation and Nutritional Determinants of Anaemia (BRINDA) project suggest that the contribution of iron deficiency as a cause of anaemia may be lower in countries with high underlying infection burden than in those with low infection burden [7]. A few reasons for the early onset of anaemia among children generally are iron deficiency, dietary history, maternal height and antenatal anaemia, along with numerous socio-economic covariates such as mother’s education, place of residence, region of residence, wealth accumulation in a family, which also have a positive association with anaemia among children [8]. From the National Family Health Survey (NFHS)-4 data, it was found that the levels of anaemia among children vary by different background factors like rural residence, lowest household wealth quintile and maternal factors and more than half of the children were suffering from mild or moderate anaemia [8].

Recently, when most developed nations are heading towards new biomedical innovations, most developing nations are still fighting problems like poverty, hunger and malnutrition. Developing and underdeveloped countries are already burdened with malnutrition and various other communicable diseases. A disproportionately higher concentration of anaemia in these countries’ accounts for about 89 percent of anaemia-related disabilities [9]. These countries are more vulnerable to anaemia as they are in the transition phase due to socio-economic changes and the emergence of global market systems [10]. A decline in anaemia was observed through the different rounds of the National Family Health Surveys from 1992–93 to 2015–16 in India. However, it is still highly prevalent as the improvements have not been equally distributed across various sub-populations in the country. India has been strengthening its resilience against the COVID-19 pandemic by prioritizing vaccine equity and addressing emerging socio-economic challenges. But NFHS-5 results indicate the reversal of the trajectory of anaemia among children, which resembles an emerging challenge for the health and wellbeing of people in the country.

Anaemia in children can limit psychomotor development and result in impaired cognitive, social, and emotional functioning, poor performance in school, and diminished productivity in their adult lives [11]. Existing literature establishes maternal anaemia as a major precursor of childhood anaemia; in view of this, the World Health Assembly set a target of achieving a 50 percent reduction of anaemia in women of reproductive age by 2025 to address the high anaemia burden across the world [12].

India, with a long history of policies and programmes to combat the prevalence of anaemia, has not seen enough improvement given India’s rapid economic growth during the same period [13]. There is a need to accelerate the reduction in anaemia and policymakers need to come up with investments that will have a major impact on the anaemia reduction in India in the future. Hence, the present study aims to decode key drivers of the reversal of trend in the prevalence of childhood anaemia between 2015–2021, coupled with emerging strategies to address this dark cloud of a health horizon in India to expedite achieving Sustainable Development Goals by 2030.

## Methods

This study utilized data from four rounds of the NFHS: NFHS-2 (1998–99), NFHS-3 (2005–06), NFHS-4 (2015–16) and NFHS-5 (2019–21). All four rounds were used to show the overall trend of anaemia among children. However, for the analysis of key drivers of the reversal of the trend of childhood anaemia, only the recent two rounds of the NFHS (NFHS-4 & NFHS-5) were used. The NFHS series is a nationally representative cross-sectional survey conducted under the stewardship of the Ministry of Health and Family Welfare (MoHFW). The survey is the Indian version of the Demographic Health Survey (DHS). The fundamental objective of this survey was to obtain state and national-level estimates on fertility, reproductive health, maternal and child health, HIV-related knowledge, infant and child mortality nutrition, family planning services, women’s autonomy, domestic violence, etc. [9]. The International Institute for Population Sciences (IIPS), Mumbai, being the nodal agency, was responsible for obtaining ethical approval for conducting and disseminating the data for the survey.

The sampling design of NFHS-5 was developed considering NFHS-4 as the benchmark and the need to provide estimates of population, health, and family welfare indicators at district, state/UT, and national levels with a reasonable level of precision.

A stratified two-stage sampling design was adopted in rural and urban areas of the 707 districts (as of March 31^st^, 2017). Within each rural stratum, villages were selected from the sampling frame using probability proportional to size (PPS) with explicit stratification based on the percentage of SC/ST population and female literacy. NFHS-5 covers 636,699 households, with 724,115 eligible women aged 15–49 and 152,752 children aged 6–59 months. The selection of households was based on the sampling frame prepared from mapping and listing households in all primary sampling units (PSUs) identified across 707 districts. NFHS-5 used four survey schedules canvassed in local languages using Computer Assisted Personal Interviewing (CAPI) and adopted numerous data quality assurance strategies to provide valid and reliable estimates of socio-demographic and biomedical indicators of population, health and development.

### Methods of anaemia estimation

Since the beginning of the NFHS, blood specimens for anaemia testing have been collected from eligible respondents by well-trained health investigators. The blood samples for haemoglobin testing were taken from the children aged 6–59 months after consent from a parent or an adult responsible for the child and from the eligible women and men after their consent. In all the rounds, a drop of the capillary blood sample drawn from a finger prick (or a heel prick for children aged 6–11 months) was used to estimate blood haemoglobin using the HemoCue Hb 201 + analyzer [13]. The HemoCue Hb 201 + analyzer is a battery-operated portable device that calibrates itself before each test and provides blood Hb levels on-site, which were immediately shared with the respondents. In each round, properly functioning machines were used. This machine was suitable for large-scale testing in field settings, easy to operate with an easy procedure of finger pricking, and very helpful in eliciting cooperation from the respondents as the test results were immediately available to them. The consistent use of capillary blood and the HemoCue Hb 201 + analyser across NFHS surveys makes it possible to monitor changes in the anaemia prevalence over time. In the NFHS, anaemia levels were estimated after adjusting for altitude and smoking as per the protocols developed by the CDC, Atlanta. Children (6–59 months) who were found to have severe anaemia (a haemoglobin level below 7 g/dl for children) were referred to a health facility for further evaluation and treatment.

The time period for capillary blood sample collection across different states/UTs of the country in NFHS-4 and NFHS-5 is given in Appendix Table A1. When collected and tested in the laboratory setup, the venous blood samples were considered the gold standard. However, the requisites of sample transport at controlled temperatures, timely delivery to laboratories, invasive nature of blood sample collection from veins, more significant complications, stringent logistic demand, and the unavailability of the results to the respondents immediately were some of the reasons why this was not preferred for the field-based investigations, and hence the capillary blood was used.

### Outcome variables

The prevalence of any anaemia among children aged 6–59 months (< 11.0 g/dl) recorded in NFHS-4 and NFHS-5 was the key outcome variable selected for the study. Variations in the prevalence of any anaemia were analysed at the national, state and district level in addition to the key drivers of the reversal of trends at individual levels.

### Predictor variables

The selected explanatory variables were categorised into three sections, namely: 1) Child level factors; 2) Maternal level factors; and 3) Socioeconomic and demographic factors. Child level factors included age of the child (in months), sex of the child and birth order. Maternal factors included mother’s education and mother’s anaemia status. Socioeconomic and demographic factors included place of residence, religion, social group and wealth quintile.

### Statistical analysis

Descriptive, bivariate and multivariable analyses were used in this study. For analysing the key drivers of childhood anaemia, any anaemia (< 11.0 g/dl) was used as the key dependent variable, regressed on a set of predictors. A binary logistic regression was used to determine the factors associated with any anaemia among children aged 6–59 months

$${\mathrm{log}}_{\mathrm{e}}\,[\mathrm{P }(\mathrm{Yi}=1|\mathrm{ Xi}) / 1-\mathrm{ P }(\mathrm{Yi}=1|\mathrm{ Xi})] =\mathrm{ loge}[\uppi |1-\uppi ] =\mathrm{ \alpha }+{\upbeta }_{1}{\mathrm{X}}_{\mathrm{i}1}\dots \dots \dots .. {\upbeta }_{\mathrm{k}} {\mathrm{X}}_{\mathrm{ik}}$$

where Yi is the binary response variable and Xi is the set of explanatory variables such as socio-demographic characteristics, and β_1_, β_2_…… β_k_ are the coefficients of the Xi variables.

Furthermore, Fairlie decomposition was used to examine the contribution of various factors to the change in the prevalence of any anaemia among children aged 6–59 months from NFHS-4 to NFHS-5. It can be expressed as follows:

$${\overline{Y} }_{u}-{\overline{Y} }_{r}=\left[\sum_{i=1}^{{N}^{u}}\frac{F\left({X}_{i}^{u}{\beta }^{u}\right)}{{N}^{u}}-\sum_{i=1}^{{N}^{r}}\frac{F\left({X}_{i}^{r}{\beta }^{u}\right)}{{N}^{r}}\right]+\left[\sum_{i=1}^{{N}^{r}}\frac{F\left({X}_{i}^{r}{\beta }^{u}\right)}{{N}^{u}}-\sum_{i=1}^{{N}^{r}}\frac{F\left({X}_{i}^{r}{\beta }^{r}\right)}{{N}^{r}}\right]$$

Where, ${\overline{Y} }_{u}$ and ${\overline{Y} }_{r}$ represent the mean value of any anaemia at two-time points ‘u’ (2015–16) and ‘r’ (2019–21), ‘X’ represents the set of predictor variables, $\beta$ represents the coefficient, ${N}^{u}$ and ${N}^{r}$ represent the sample size at time points u and r, respectively. First term in the equation represents characteristics and latter term represents the discrimination effect, that is, the differences caused by various characteristic regression coefficients. Positive sign represents higher contribution to the difference in NFHS-5 as compared to NFHS-4 and vice-versa.

## Results

**Fig. 1:**
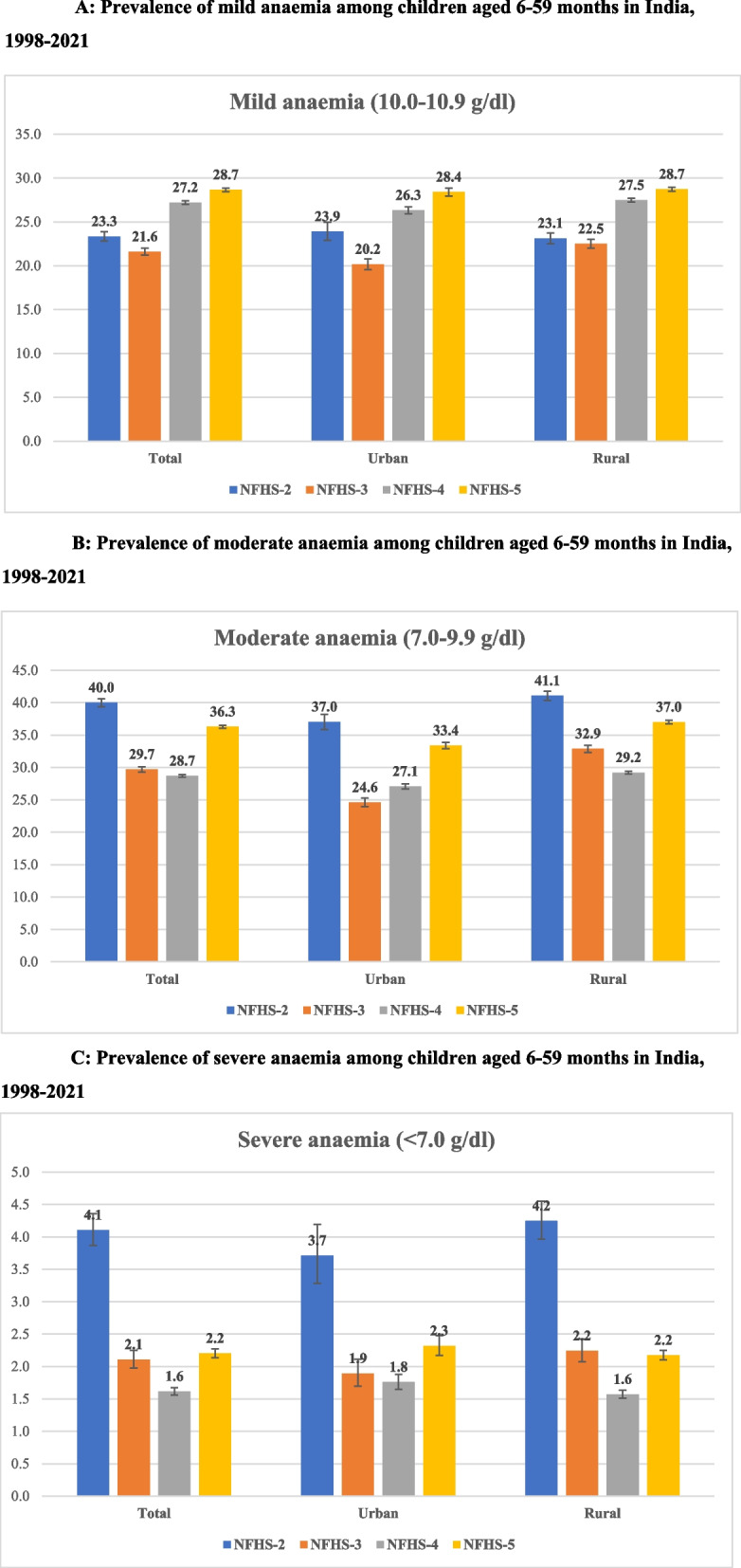
**A** Prevalence of mild anaemia among children aged 6–59 months in India, 1998–2021. **B** Prevalence of moderate anaemia among children aged 6–59 months in India, 1998–2021. **C** Prevalence of severe anaemia among children aged 6–59 months in India, 1998–2021

The prevalence of mild anaemia (10–10.9 g/dl) increased from 23 percent in NFHS-2 to 29 percent in NFHS-5 (Fig. 1A). However, the prevalence of moderate or severe anaemia (< 10 g/dl) declined considerably from NFHS-2 to NFHS-4 and suddenly showed an increase in NFHS-5 (Fig. 1B & C).

To investigate the distribution of haemoglobin levels between surveys, a kernel density curve was produced using the haemoglobin levels of children aged 6–59 months from NFHS-4 and NFHS-5 (Appendix Figure A1). The result showed a similar distribution across the surveys.

Table 1 presents the prevalence of anaemia among children aged 6–59 months from the two recent rounds of the NFHS. In NFHS-4, Mizoram (19.3%) showed the lowest prevalence, while the state of Haryana (71.7%) reported the highest prevalence. However, in NFHS-5, the highest prevalence was found in Gujarat (79.7%), followed by Jammu & Kashmir (72.7%) and Madhya Pradesh (72.7%) and the lowest in Kerala with a prevalence of 39.4 percent. Talking about the state-specific changes in the prevalence of childhood anaemia, a substantial increase was found in 25 out of 29 states. To assess the significance of these changes, 95% confidence intervals in this estimated prevalence of any anaemia for both NFHS-4 & 5 were presented, which confirmed a significant increase in the prevalence of childhood anaemia in the majority of the states. On the other hand, only four states (Meghalaya, Jharkhand, Haryana, and Uttrakhand) demonstrated a substantial fall (*p* < 0.001) in the prevalence, with the range of the decrease being between 1 and 2.9 percent in Meghalaya and Uttrakhand.

**Table 1:** Percentage of children aged 6–59 months having anaemia and confidence interval (CI) by state, National Family Health Survey, 2016–21

**State**	**NFHS-4 (2015–16)**		**NFHS-5 (2019–2021)**		Difference	*p*- value
	**Percent**	**CI**	**Percent**	**CI**		
Andhra Pradesh	58.6	56.1–61.1	63.2	60.7–65.6	4.6	*p* = 0.002
Arunachal Pradesh	54.2	52.5–56.2	56.6	53.9–59.2	2.4	*p* = 0.029
Goa	48.3	41.3–55.4	53.2	46.1–60.3	4.9	*p* = 0.221
Haryana	71.7	70.1–73.4	70.4	68.8–72.0	-1.3	*p* < 0.001
Himachal Pradesh	53.7	50.1–57.3	55.4	52.5–58.2	1.7	*p* = 0.253
Jharkhand	69.9	68.7–71.2	67.5	65.9–69.0	-2.4	*p* < 0.001
Kerala	35.7	32.5–38.7	39.4	36.5–42.3	3.7	*p* = 0.012
Sikkim	55.1	49.8–60.3	56.4	48.7–64.2	1.3	*p* = 0.651
Uttarakhand	59.8	58.5–61.9	58.8	55.9–61.7	-1	*p* < 0.001
Gujarat	62.6	60.6–64.5	79.7	78.2–81.1	17.1	*p* < 0.001
Meghalaya	48.0	46.3–49.9	45.1	42.1–48.1	-2.9	*p* < 0.001
Tamil Nadu	50.7	48.5–52.3	57.4	55.8–59.1	6.7	*p* < 0.001
Telangana	60.7	57.7–63.8	70.0	68.3–71.8	9.3	*p* < 0.001
Tripura	48.3	44.6–52.0	64.2	61.1–67.3	15.9	*p* < 0.001
Assam	35.7	34.1–37.2	68.4	66.7–70.1	32.7	*p* < 0.001
Bihar	63.5	62.6–64.4	69.4	68.3–70.6	5.9	*p* < 0.001
Chhattisgarh	41.6	39.8–43.3	67.2	65.4–69.1	25.6	*p* < 0.001
Delhi	59.7	53.6- 65.8	69.2	66.7–71.7	9.5	*p* < 0.001
Jammu & Kashmir	53.8	52.6- 55.7	72.7	70.2–75.1	18.9	*p* < 0.001
Karnataka	60.9	58.9–62.8	65.5	63.7–67.3	4.6	*p* < 0.001
Madhya Pradesh	68.9	68.1–69.8	72.7	71.4–73.9	3.8	*p* < 0.001
Maharashtra	53.8	51.9–55.7	68.9	67.3–70.5	15.1	*p* < 0.001
Manipur	23.9	22.6- 25.4	42.8	40.0–45.5	18.9	*p* < 0.001
Mizoram	19.3	17.1–21.2	46.4	42.7–50.0	27.1	*p* < 0.001
Nagaland	26.4	25.0- 28.3	42.7	39.5–46.0	16.3	*p* < 0.001
Odisha	44.6	43.1–46.0	64.2	62.7–65.8	19.6	*p* < 0.001
Punjab	56.6	54.3–59.0	71.1	69.2–73.0	14.5	*p* < 0.001
Rajasthan	60.3	59.1–61.5	71.5	70.1–72.8	11.2	*p* < 0.001
Uttar Pradesh	63.2	62.4–63.9	66.4	65.5–67.3	3.2	*p* < 0.001
West Bengal	54.2	52.1–56.2	69	67.0–71.0	14.8	*p* < 0.001
**India**	**58.5**	**58.2–58.9**	**67.1**	**66.7–67.5**	8.6	*p* < 0.001

**Fig. 2:**
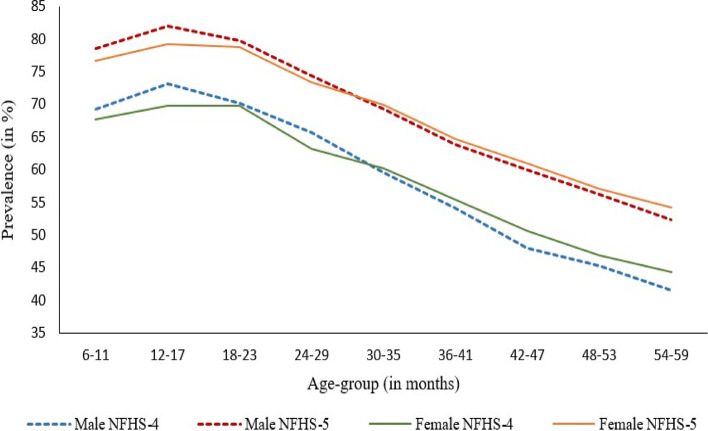
Age-sex specific prevalence of anaemia among children aged 6–59 months in NFHS-4, & NFHS-5

Figure 2 presents the age-sex specific prevalence of anaemia among children aged 6–59 months over the last two rounds of the NFHS in India (Appendix Table A2). There was an almost ten percentage point increase in the prevalence, reflecting a uniform pattern in the six-month age groups. The prevalence of anaemia among children increased with age and peaked at the ages of 12–17 months for male babies (72.35% in NFHS-4 & 81.74% in NFHS-5) and 18–23 months for female babies (69.61% in NFHS-4 & 79.17% in NFHS-5) and then showed a declining pattern. Despite the declining pattern, the prevalence of anaemia remained higher among male babies than in females up to 30 months of age. After attaining the age of 30 months, which is the peak age for supplementary feeding among Indian children, the pattern of sex differentials in anaemia prevalence got reversed, with a relatively higher prevalence among females than males. Females remained at a disadvantage of being anaemic than their male counterparts in the age group of 30–59 months. The pattern remained the same in NFHS-4 and in NFHS-5.

**Fig. 3:**
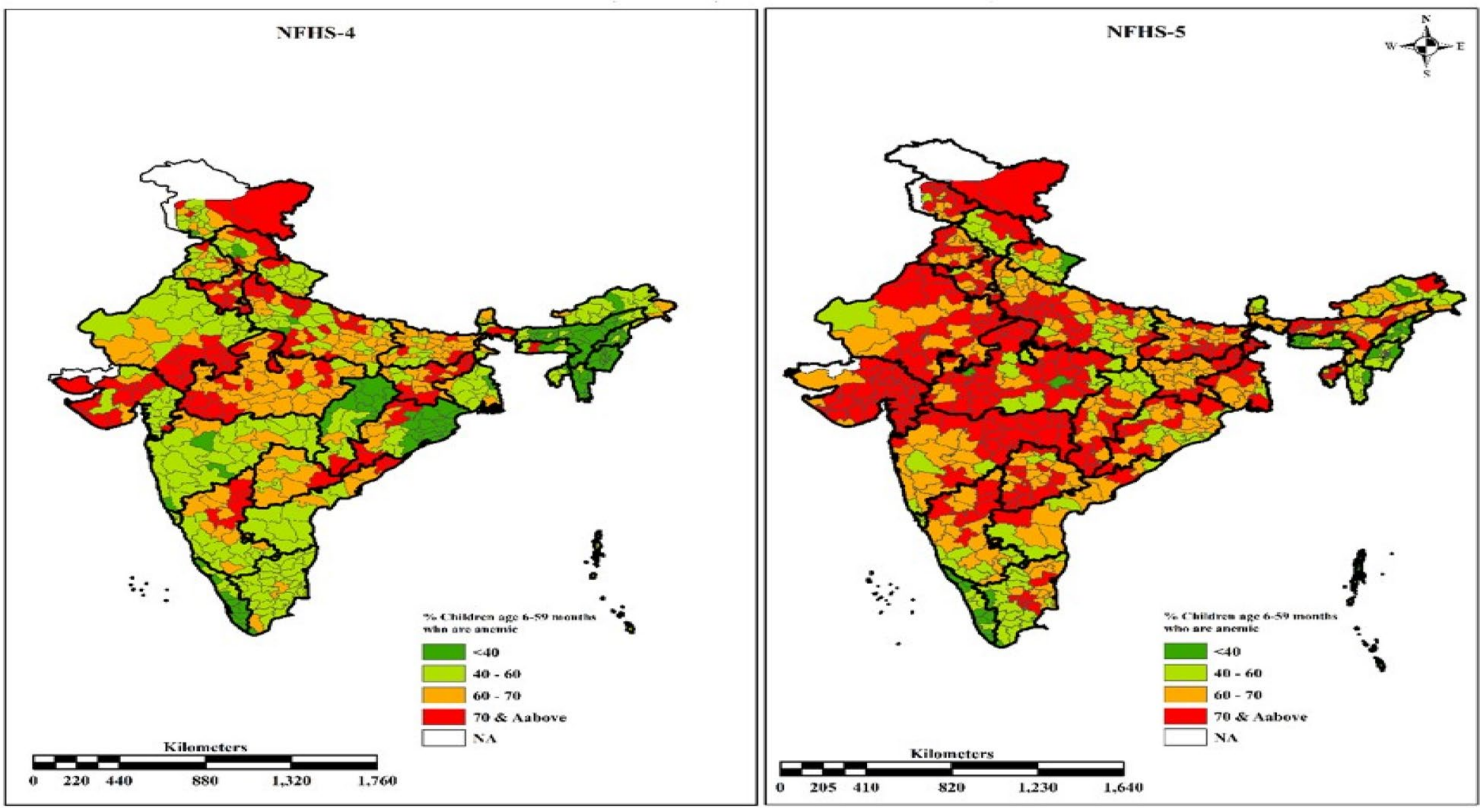
Changes in prevalence of anaemia among children age 6–59 months from NFHS-4 (2015–16) to NFHS-5 (2019–20). Source: Created using ArcGIS Pro 2.6 (https://arcgis.pro/download-arcgis-pro-2-6-for-free/)

Figure 3 portrays the variation in the prevalence of anaemia among children aged 6–59 months in various districts of India in NFHS-4 and 5. The prevalence of any anaemia (< 11.0 g/dl) increased in many districts, cutting across the development levels of states. According to NFHS-4 (2015–16), 213 out of 640 districts in India had at least 65 percent of their youngsters anaemic, which rose to 61 percent (434 out of 707) in NFHS-5.The number of districts with more than 65 percent of anaemic children had increased all over India, especially in the Central and Western regions. There was a substantial decline in the number of districts in the country with less than 50 percent of anaemic children, except for some areas in the North-eastern region.

The results in Table 2 portray a wide range of variations in the prevalence of anaemia among the children aged 6–59 months by the different background characteristics in the two recent rounds of the Indian DHS. Most of the background characteristics included in the analysis showed significant variation across various categories of predictors and an increasing prevalence of anaemia from NFHS-4 to NFHS-5. A uniform increase of 7–9 percentage points from NFHS-4 to NFHS-5 was observed in the prevalence of anaemia among the children in each age group, both male and female children and those belonging to rural and urban areas (Table 2). The prevalence of anaemia among children aged 6–59 months increased with the increasing birth order in both rounds of the NFHS. There was also a significant increase in the prevalence of anaemia across both rounds among the children with birth order 2–3 (56% in NFHS-4 to 68% in NFHS-5), birth order 4–5 (60% in NFHS-4 to 70% in NFHS-5) and among children with birth order 6 or more (64% in NFHS-4 to 71% in NFHS-5).

**Table 2:** Percentage of children aged 6–59 months classified as having anaemia (Hb < 11.0 g/dl) by background characteristics, National Family Health Survey, 2016–21

**Background characteristic**	**NFHS-4**			**NFHS-5**			Difference	*p*- value
	Prevalence	Number of children	*p*- value	Prevalence	Number of children	*p*- value		
**Child’s Age (in months)**			*p* < 0.001			*p* < 0.001		
6–8	68.4	10271		75.2	7998		6.8	*p* < 0.001
9–11	68.6	10980		78.7	8566		10.1	*p* < 0.001
12–17	71.2	22607		80	17107		8.8	*p* < 0.001
18–23	69.9	22842		78.2	16391		8.3	*p* < 0.001
24–35	62.3	45188		70.5	34155		8.2	*p* < 0.001
36–47	52.3	47601		61	33825		8.7	*p* < 0.001
48–59	44.7	45547		53.4	34711		8.7	*p* < 0.001
**Sex of the Child**			*p* = 0.862			*p* = 0.322		
Male	58.4	106802		67.2	79515		8.8	*p* < 0.001
Female	58.7	98233		67	73237		8.3	*p* < 0.001
**Birth order**			*p* < 0.001			*p* < 0.001		
1	55.6	75222		65.6	61967		10	*p* < 0.001
2–3	59.5	94191		67.7	75806		8.2	*p* < 0.001
4–5	63.7	21369		70.4	12119		6.7	*p* < 0.001
6 or more	64.7	7069		71.2	2324		6.5	*p* < 0.001
**Mother’s education**			*p* < 0.001			*p* < 0.001		
No schooling	64.9	61867		71.4	22223		6.5	*p* < 0.001
< 5 years complete	60	12247		70	7432		10	*p* < 0.001
5–7 years complete	58.7	32632		70.3	23497		11.6	*p* < 0.001
8–9 years complete	56.6	33181		68.5	31030		11.9	*p* < 0.001
10–11 years complete	55.1	23756		66.1	22747		11	*p* < 0.001
12 or more years complete	51.7	37085		62.5	45822		10.8	*p* < 0.001
**Mother’s anaemia status**			*p* < 0.001			*p* < 0.001		
Not anaemic	50.6	85663		60	61920		9.4	*p* < 0.001
Mildly anaemic	62.3	83309		68.7	40152		6.4	*p* < 0.001
Moderately anaemic	71.3	27990		74.6	46149		3.3	*p* < 0.001
Severely anaemic	75.7	1885		77.1	3281		1.4	*p* = 0.253
**Residence**			*p* < 0.001			*p* < 0.001		
Urban	56	56237		64.2	42917		8.2	*p* < 0.001
Rural	59.5	148798		68.3	109835		8.8	*p* < 0.001
**Religion**			*p* < 0.001			*p* < 0.001		
Hindu	58.7	160878		67.5	122437		8.8	*p* < 0.001
Muslim	59.5	34233		66.8	23317		7.3	*p* < 0.001
Christian	44.8	4149		53.1	3441		8.3	*p* < 0.001
Sikh	56.3	2632		70.3	1940		14	*p* < 0.001
Buddhist/Neo-Buddhist	57	1622		71.1	815		14.1	*p* < 0.001
Jain	53	220		72.3	252		19.3	*p* < 0.001
Other	68.4	1302		67	550		-1.4	*p* < 0.001
**Social group**			*p* < 0.001			*p* < 0.001		
Scheduled Castes	60.6	45208		69.5	36135		8.9	*p* < 0.001
Scheduled Tribes	63.3	21411		72.4	14481		9.1	*p* < 0.001
Other backward Class	58.6	90237		65.2	65009		6.6	*p* < 0.001
Other	54.2	46649		65.8	35749		11.6	*p* < 0.001
Don’t know	61.9	1530		73.3	1379		11.4	*p* < 0.001
**Wealth quintile**			*p* < 0.001			*p* < 0.001		
Lowest	64	52483		71.5	30961		7.5	*p* < 0.001
Second	59.7	45355		69.3	32889		9.6	*p* < 0.001
Middle	58.9	40598		67.1	32072		8.2	*p* < 0.001
Fourth	54.4	37100		64.2	30741		9.8	*p* < 0.001
Highest	51.8	29500		62.6	26089		10.8	*p* < 0.001
**Total**	**58.5**	**205035**		**67.1**	**152752**		8.6	*p* < 0.001

Among the different social (caste) groups, Scheduled Tribe (63%) had the highest prevalence of anaemic children as per the fourth round of the NFHS, followed by Scheduled Castes (61%), and Other Backward Classes (59%). A similar pattern was found in the NFHS-5. With the increasing educational attainment of mothers, there was a decline in the likelihood of being anaemia among the children. Variation in the prevalence of childhood anaemia by the wealth quintile portrayed a significant increase (*p* < 0.001) among the children aged 6–59 months from the lowest quintile to the highest quintile, observed in both NFHS-4 and NFHS-5. The increase in prevalence across both rounds was seen to be the least among the children belonging to the lowest wealth quintile, with an increase of 7.5 percentage points, and the maximum increase was seen among the ones from the highest quintile (10.8 percentage points).

Variation in the prevalence of childhood anaemia by different categories of mother’s anaemia status over two rounds of NFHS revealed a ten percentage point increase (from 51% in NFHS-4 to 60% in NFHS-5) (*p* < 0.001) in anaemia prevalence with mothers who were not anaemic. This differential increase across different levels of the mother’s anaemia portrayed a constantly higher prevalence in NFHS-5 than among children of mothers recorded in NFHS-4 with the increase in the severity of the mother’s anaemia status.

Table 3 shows the prevalence of different levels of anaemia among children aged 6–59 months by the background characteristics for NFHS-4 and NFHS-5. Results envisage that the prevalence of all three levels of anaemia increased from NFHS-4 to NFHS-5. The percentage increase in the levels of severe anaemia (31%) was higher than the level of increase in mild (5%) and moderate (22.6%) anaemia between both the rounds. In both rounds, mild anaemia (10–10.9 g/dl) was less common in boys than in girls. Children with birth orders of six or more had a greater prevalence of mild, moderate, and severe anaemia. In comparison to mild and severe anaemia, there was a greater variation in the levels of moderate anaemia between urban and rural areas. Mild and moderate anaemia were higher among children in rural areas, and severe anaemia was higher among those from urban areas. NFHS-5 data further showed that anaemia among children reduces with age, especially after 24 months at different levels of development. The prevalence among the children in the younger age groups (less than 24 months) was higher than the state average. The prevalence was lowest in the age group of 45–59 months.

**Table 3:** Percentage of children aged 6–59 months having a different level of Anaemia by their background characteristics in India, NFHS-4 & NFHS-5

**Background Characteristics**	**Mild (10.0–10.9 g/dl)**		**Moderate (7.0–9.9 g/dl)**		**Severe (< 7.0 g/dl)**		*p*-value	
	**NFHS-4 (%)**	**NFHS-5 (%)**	**NFHS-4 (%)**	**NFHS-5 (%)**	**NFHS-4 (%)**	**NFHS-5 (%)**	**NFHS-4 (%)**	**NFHS-5 (%)**
**Child’s Age (in months)**							*p* < 0.001	*p* < 0.001
6–8	30.1	29.0	37.0	44.2	1.4	2.0		
9–11	28.1	27.5	38.4	48.1	2.1	3.1		
12–17	27.7	26.3	41.0	50.3	2.4	3.5		
18–23	27.4	27.9	39.7	46.8	2.8	3.5		
24–35	28.6	29.4	31.9	38.7	1.8	2.4		
36–47	28.1	31.0	23.2	28.6	1.1	1.4		
48–59	26.4	30.0	17.6	22.6	0.7	0.8		
**Sex of the Child**							*p* < 0.001	*p* = 0.001
Male	27.3	29.0	29.5	36.0	1.6	2.2		
Female	28.4	29.5	28.8	35.6	1.5	2.0		
**Birth order**							*p* < 0.001	*p* < 0.001
1	27.8	29.5	26.5	34.1	1.3	1.9		
2–3	28.0	28.9	29.9	36.7	1.6	2.2		
4–5	27.4	29.8	34.3	38.3	2.0	2.3		
6 or more	27.7	30.1	34.7	38.5	2.3	2.6		
**Mother’s education**							*p* < 0.001	*p* < 0.001
No schooling	28.7	29.7	34.3	39.3	1.9	2.4		
< 5 years complete	28.2	29.4	30.2	38.4	1.6	2.2		
5–7 years complete	28.3	29.6	28.9	38.3	1.5	2.4		
8–9 years complete	27.3	29.8	27.8	36.6	1.4	2.1		
10–11 years complete	27.6	28.7	26.3	35.5	1.2	1.9		
12 or more years complete	26.5	28.7	24.0	32.0	1.3	1.8		
**Mother’s anaemia status**							*p* < 0.001	*p* < 0.001
Not anaemic	26.6	28.6	22.9	29.8	1.1	1.5		
Mildly anaemic	29.3	30.6	31.4	36.1	1.5	2.0		
Moderately anaemic	27.6	29.3	41.0	42.6	2.8	2.7		
Severely anaemic	22.6	23.2	46.3	48.0	6.8	5.9		
**Residence**							*p* < 0.001	*p* < 0.001
Urban	26.8	29.0	27.5	33.1	1.6	2.2		
Rural	28.2	29.3	29.8	36.9	1.5	2.1		
**Religion**							*p* < 0.001	*p* < 0.001
Hindu	27.8	29.0	29.3	36.3	1.6	2.1		
Muslim	27.9	30.8	29.9	34.3	1.7	1.8		
Christian	25.3	26.6	18.6	25.0	0.9	1.5		
Sikh	27.5	25.7	27.6	39.8	1.2	4.8		
Buddhist/Neo-Buddhist	27.6	30.9	27.8	36.7	1.6	3.5		
Jain	30.5	39.7	22.1	28.4	0.4	4.3		
Other	35.3	29.2	32.6	36.0	0.4	1.8		
**Social group**							*p* < 0.001	*p* < 0.001
Scheduled Castes	28.2	29.2	30.7	37.8	1.6	2.5		
Scheduled Tribes	28.5	29.8	33.3	40.4	1.5	2.2		
Other backward Class	27.8	28.7	29.2	34.6	1.6	1.9		
Other	27.2	30.1	25.6	33.7	1.4	1.9		
Don’t know	27.7	28.7	32.4	43.2	1.8	1.4		
**Wealth quintile**							*p* < 0.001	*p* < 0.001
Lowest	29.2	30.1	33.3	39.6	1.5	1.9		
Second	28.5	30.0	29.7	37.2	1.6	2.1		
Middle	27.8	29.2	29.3	35.7	1.8	2.2		
Fourth	26.2	28.1	26.8	33.8	1.4	2.3		
Highest	26.5	28.6	24.0	31.9	1.4	2.0		
**Total**	27.8	29.2	29.2	35.8	1.6	2.1		

Two crucial maternal traits, the mother's years of education and her anaemia status, have a direct influence on the child's anaemia status, as the earlier findings had also shown. With the increase in the number of years of mother's schooling, anaemia among children decreased in both NFHS-4 and NFHS-5. Table 3 also showed an increase in the percentage of children with mild, moderate, and severe anaemia with the rise in the mother's anaemic status from NFHS-4 to NFHS-5, which indicates that anaemia was much more prevalent among the offspring of anaemic mothers.

Table 4 presents the results of the logistic regression model showing the association between anaemia and different background characteristics. Maternal factors like mother’s education and mother’s anaemia status showed a significant association with the anaemia status of the children. Accordingly, the results show that maternal education was negatively associated with childhood anaemia as children of educated mothers showed a lesser odd of anaemia than the children of uneducated mothers, both in NFHS-4 and NFHS-5. Furthermore, children of severely anaemic mothers were three times (AOR-3.143; 95% CI:2.826–3.495) and two times (AOR-2.549; 95% CI:2.359–1.753) more likely to be anaemic than the children of non-anaemic mothers in NFHS-4 and NFHS-5, respectively. In the case of the socio-economic characteristics also, children belonging to Schedule Tribe were 13 percent (AOR-1.129; 95% CI:1.087–1.173) (NFHS-5) significantly more likely to be anaemic as compared to the children belonging to Schedule Caste. However, children of Other Backward Classes and Other caste groups were significantly less likely to be anaemic than children of the Schedule Caste. In addition, the wealth index also showed a significant negative association with childhood anaemia.

**Table 4:** Logistic regression estimates of any anaemia (Hb < 11.0 g/dl) by background characteristics among children aged 6–59 months

**Background characteristic**	**NFHS-4**		**NFHS-5**	
	AOR	95% CI	AOR	95% CI
**Child’s Age (in months)**				
6–8				
9–11	1.097^**^	1.034–1.164	1.194^***^	1.111–1.283
12–17	1.210^***^	1.148–1.274	1.292^***^	1.213–1.376
18–23	1.098^***^	1.043–1.157	1.135^***^	1.066–1.209
24–35	0.749^***^	0.715–0.785	0.756^***^	0.714–0.800
36–47	0.496^***^	0.473–0.520	0.499^***^	0.471–0.528
48–59	0.372^***^	0.355–0.390	0.362^***^	0.342–0.383
**Sex of the Child**				
Male				
Female	0.998	0.980–1.017	0.988	0.968–1.009
**Birth order**				
1				
2–3	1.056^***^	1.034–1.078	1.028^*^	1.005–1.051
4–5	1.073^***^	1.038–1.108	1.02	0.981–1.060
6 or more	1.069^*^	1.015–1.126	1.004	0.939–1.074
**Mother’s education**				
No schooling				
< 5 years complete	0.799^***^	0.767–0.832	0.897^***^	0.853–0.943
5–7 years complete	0.816^***^	0.791–0.840	0.882^***^	0.851–0.914
8–9 years complete	0.721^***^	0.700–0.743	0.805^***^	0.777–0.833
10–11 years complete	0.708^***^	0.683–0.734	0.759^***^	0.730–0.790
12 or more years complete	0.638^***^	0.616–0.661	0.676^***^	0.652–0.702
**Mother’s anaemia status**				
Not anaemic				
Mildly anaemic	1.682^***^	1.649–1.716	1.454^***^	1.418–1.491
Moderately anaemic	2.591^***^	2.515–2.670	1.966^***^	1.917–2.016
Severely anaemic	3.143^***^	2.826–3.495	2.549^***^	2.359–1.753
**Residence**				
Urban				
Rural	0.973	0.949–0.997	1.027	0.998–1.057
**Religion**				
Hindu				
Muslim	1.043^**^	1.015–1.072	1.029	0.997–1.063
Christian	0.383^***^	0.368–0.400	0.379^***^	0.363–0.396
Sikh	1.027	0.955–1.105	1.096^*^	1.010–1.191
Buddhist/Neo-Buddhist	1.147^**^	1.042–1.264	1.189^**^	1.067–1.326
Jain	0.939	0.694–1.270	1.620^*^	1.112–2.360
Other	0.769^***^	0.707–0.837	0.553^***^	0.506–0.605
**Social group**				
Scheduled Castes				
Scheduled Tribes	1.045^*^	1.010–1.081	1.129^***^	1.087–1.173
Other backward Class	0.946^***^	0.921–0.972	0.886^***^	0.860–0.912
Other	0.806^***^	0.782–0.832	0.899^***^	0.868–0.930
**Wealth quintile**				
Lowest				
Second	0.922^***^	0.897–0.948	0.949^**^	0.920–0.978
Middle	0.938^***^	0.910–0.967	0.955^**^	0.923–0.987
Fourth	0.906^***^	0.876–0.938	0.928^***^	0.894–0.963
Highest	0.952^***^	0.914–0.992	0.916^***^	0.876–0.957
**Cons**	2.160^***^	**2.034–2.293**	3.339^***^	**3.107–3.588**

Note: *,** and *** referes to 0.05, 0.01 and 0.001 level of significance, respectively

The results of Fairlie decomposition, applied to assess the contribution of various factors to the change in the prevalence of anaemia among children aged 6–59 months, are presented in Table 5. According to the results, except for the sex of the child, all other variables were found to be significantly contributing to the change in the prevalence of anaemia. In the case of child’s age, the negative percent values (-7.411) indicate that child's age contributed negatively to the overall change in the anaemia prevalence among children, which means the effect of child’s age on the prevalence of anaemia was lower in NFHS-5 as compared to NFHS-4. Likewise, factors like birth order, mother’s education, and place of residence were found to be negatively contributing to the change in childhood anaemia. Nevertheless, factors like mother’s anaemia status (28.239) was found to be significantly positively contributing to the overall change in the anaemia prevalence. In addition, factors like caste, religion and wealth quintile were positive contributors to the change in the prevalence of childhood anaemia.

**Table 5:** Fairlie decomposition of any anaemia among children aged 6–59 months

Difference	-0.0962			
Total explained	0.0107			
**Variable**	**Coefficient**	**95% CI**		**% Contribution**
Child’s Age	0.0071^***^	0.0069	0.0073	-7.411
Sex of the Child	0.0000	0.0000	0.0000	-0.003
Birth order	0.0001^*^	0.000	0.0002	-0.120
Mother’s education	0.0094^***^	0.0088	0.0100	-9.786
Mother’s anaemia status	0.0272^***^	0.0278	-0.0265	28.239
Residence	0.0004^***^	0.0002	0.0007	-0.447
Religion	0.0002^***^	0.0002	-0.0002	0.173
Social group	0.0003^***^	0.0004	-0.0001	0.264
Wealth quintile	0.0002^***^	0.0003	-0.0001	0.181

Note: *,** and *** refer to 0.05, 0.01 and 0.001 level of significance, respectively

## Discussion

The findings from this study portray that the prevalence of anaemia has increased among children aged 6–59 months over the last four years. This change is primarily driven by factors like mother’s educational level, mother’s anaemia status, socio-economic status etc. The findings also suggest that the increase in the prevalence of any anaemia is not uniform across states. Hence, indicating the interplay of both regional and local heterogeneity with diverse ingredients, which need to be explored by small-scale qualitative exploratory studies along with key insights from programme personnel. Many malaria-prone states like Madhya Pradesh, and Jharkhand exhibit a high prevalence of anaemia [14].

Over the years, the Government of India launched many programmes to reduce anaemia level among vulnerable populations by improving their nutritional status. In 1970, India became the first developing nation to launch a nationwide anaemia programme named the National Nutritional Anaemia Prophylaxis Project (NNAPP). Through this programme, Iron and Folic Acid (IFA) tablets were implemented and distributed to children, pregnant and lactating women at the health facilities. In 1991, the programme was redesigned as the National Nutritional Anaemia Control Program [2]. The programme was originally intended to be implemented as a part of the Reproductive and Child Health (RCH), but it is now a part of the Integrated Child Development Scheme (ICDS). In order to enhance the nutritional condition of pre-schoolers, particularly those from underprivileged or underdeveloped areas, the Indian government started the ICDS programme. Some studies have suggested that the IFA tablets given to children (below three years) were not easily acceptable and recommended liquid IFA for young children [15]. Therefore, the government changed its strategy in 2007 and switched from tablets to liquid IFA. According to the new policy, young children (6–59 months) get one milliliter of IFA syrup for 100 days in a year that contains 20 mg elemental iron and 100 μg folic acid [16]. However, these micronutrient supplementation programmes primarily target those with severe anaemia, which can explain the trend reduction in moderate and severe anaemia but not in mild anaemia.

This study highlights a few key drivers of the reversal of the trajectory of childhood anaemia in India. Maternal anaemia has been the major factor behind the prevalence of anaemia in children aged 6–59 months. Maternal anaemia has also been found as a significant key driver of the change in the prevalence of anaemia. This association of a mother’s anaemic status with the prevalence of anaemia in children is also supported by a multilevel study done on four Southern African countries [17]. A possible explanation could be that the mothers with anaemia reside in poor households and might have problems providing nutritious food for themselves and their children, leading to inadequate intake of iron and other micronutrients, causing anaemia.

The prevalence of anaemia among the children is observed to be decreasing with the increase in the level of education of the mother. A study done on Korean school-aged children also support that the children with more highly educated mothers were less likely to suffer from anaemia and iron deficiency than the children with less-educated mothers [18]. Higher education results in a greater understanding of health and nutrition, which can improve the standard of the diet that children consume.

Despite the above observations, it may seldom precisely explain the reversal of the trajectory in childhood anaemia in the NFHS-5 results, as anaemia has many biomedical, social, behavioural, and environmental causes. In India and globally, about half of the anaemic cases are due to nutritional deficiencies. Other mechanisms that result in anaemia are the decreased or faulty production of red blood cells, the reduced life span of red blood cells, blood loss, etc. It can also be caused by micronutrient deficiencies like folic acid, Vitamin B-12, haemoglobinopathies, malaria, hookworms, other helminths, chronic infections, and genetic conditions [19]. If any of these underlying factors change over time, it can likely change the prevalence of anaemia in one direction or the other. The timing of the fieldwork in each NFHS survey round is another important factor influencing the prevalence of anaemia. The prevalence of anaemia would probably change if the fieldwork was carried out in various seasons in several states in NFHS-4 and NFHS-5 since nutritional anaemia, malarial anaemia, and infections have seasonal peaks and troughs.

Many other studies have highlighted that a lack of millets in the diet due to overdependence on rice and wheat, insufficient consumption of green and leafy vegetables, and the dominance of packaged and processed foods that are low in nutrition could be the reasons behind the high prevalence of anaemia in India [20, 21]. An article on the determinants of childhood anaemia in India indicated a dearth of published literature describing the drivers of childhood anaemia in children less than five years in India [22]. Some of the significant effects of climate change are impairments of crop and agricultural yields, food shortages and insecurity, a challenge to household wealth, and childhood anaemia, leading to climate change also being an essential driver of anaemia [23]. The study by Sharma et al. [10] analysed the spatial heterogeneity in the prevalence of anaemia using autocorrelation and autoregression models. The results showed that anaemia affected more than 65 percent of children aged 6–59 months in over 33 percent of Indian districts. The results of the Spatial Error Model explained that the coefficients of mother's anaemia (0.74), uneducated mothers (0.10), and underweight children (0.10) were some of the critical predictors of childhood anaemia in India.

NFHS-5 results show that the prevalence of anaemia among children continues to be a significant public health problem despite its resonance in most of the population and health indicators. It would be worthwhile to conduct small-scale exploratory studies at a regional level to better understand the impact of the COVID situation on the reversal of the trajectory of childhood anaemia to effectively tackle and curtail this public health issue. A big part of the NFHS-5 fieldwork for most of the phase two states was conducted during post COVID-19 lockdown. The impact of the pandemic situation on socio-economic and biological factors might also have increased the risk of developing anaemia in the population at large. The ground reality of the COVID-19 outbreak in multiple waves impacts the accessibility of basic amenities and services like water, sanitation, hygiene, food supplies, loss of children’s health, including their anaemia levels.

## Conclusions

Factors such as maternal education, anaemia status of mother, socio-economic status, etc. were found to be the key drivers of the change in the prevalence of childhood anaemia between the last two NFHS surveys. Understanding their contributions may help strengthen the existing programmes/schemes like Poshan Abhiyaan, *Anaemia Mukt Bharat, *etc. There is an urgent need for a more stringent technology-based monitoring mechanism for the food supplementation programme in India in order to reduce the level of childhood anaemia. Also, a focus is needed on improving women’s education so that they can make better dietary choices for themselves and their children. Moreover, creating a livelihood for low-income households to strengthen their economic condition can aid in enhancing children's nutrition. Convergence in various nutrition-sensitive interventions and triangulation in service delivery may be ensured even in a pandemic situation by adopting innovative approaches for various interventions’ efficiency and effectiveness to improve the country’s situation. Further, the Anaemia Mukt Bharat programme should also include area-specific diverse components to address nutritional anaemia, sickle cell anaemia, and various local and regional infections like hookworms, beyond iron supplementation among pregnant women and children, with special focus on socially deprived and economically marginalized groups.

### Supplementary Information

**Additional file 1: Table A1.** Time period for capillary blood sample collection across states/UTs, NFHS-4 & NFHS-5. **Figure A1.** Kernal density curve showing the distribution of haemoglobin level in NFHS4 and NFHS-5. **Table A2.** Age-sex specific prevalence of anaemia among children aged 6-59 months in NFHS-4, & NFHS-5.

## Data Availability

The study utilizes data from a national survey conducted under the stewardship of Ministry of Health & Family Welfare, Government of India, with the help of International Institute for Population Sciences, Mumbai. The data has been archived in the public repository of the Demographic and Health Survey of India. The data can be accessed using the link: https://www.dhsprogram.com/data/available-datasets.cfm.
