# Supplementary material for: Key drivers of reversal of trend in childhood anaemia in India: evidence from Indian demographic and health surveys, 2016–21
Source: BMC Public Health. 2023 Aug 18;23:1574. doi: 10.1186/s12889-023-16398-w (PMC10436448; doi:10.1186/s12889-023-16398-w)
Supplement: Supplementary file 1 — Additional file 1: Table A1. Time period for capillary blood sample collection across states/UTs, NFHS-4 & NFHS-5. Figure A1. Kernal density curve showing the distribution of haemoglobin level in NFHS4 and NFHS-5. Table A2. Age-sex specific prevalence of anaemia among children aged 6-59 months in NFHS-4, & NFHS-5. [file 12889_2023_16398_MOESM1_ESM.docx]

**Appendix**

**Table A1:** **Time period for capillary blood sample collection across states/UTs, NFHS-4 & NFHS-5**

| **NFHS-4** | **NFHS-4 (Month and year of fieldwork)** | **NFHS-5 (Month and year of fieldwork)** |
| --- | --- | --- |
| **North** |  |  |
| Chandigarh | May, 2016 to June, 2016 | March, 2021 to April, 2021 |
| Delhi | February, 2016 to September, 2016 | January 2020 to March 2020 **&** November, 2020 to January, 2021 |
| Haryana | February, 2015 to June, 2015 | January, 2020 to March 2020 **&** December, 2020 to April, 2021 |
| Himachal Pradesh | February, 2016 to August, 2016 | July, 2019 to November, 2019 |
| Jammu and Kashmir | January, 2016 to November, 2016 | July, 2019 to January, 2020 |
| Ladakh |  | August, 2019 to September, 2019 |
| Punjab | January, 2016 to June, 2016 | January, 2020 to March, 2020 **&** December, 2020 to March, 2021 |
| Rajasthan | January, 2016 to July, 2016 | January, 2020 to March, 2020 **&** December, 2020 to March, 2021 |
| Uttarakhand | January, 2015 to July, 2015 | December, 2020 to March, 2021 |
| **Central** |  |  |
| Chhattisgarh | January, 2016 to June, 2016 | January, 2020 to March, 2020 **&** December, 2020 to March, 2021 |
| Madhya Pradesh | January, 2015 to July, 2015 | January, 2020 to March,2020 **&** November, 2020 to April, 2021 |
| Uttar Pradesh | January, 2015 to September, 2016 | January, 2020 to March, 2020 **&** November, 2020 to April, 2021 |
| **East** |  |  |
| Bihar | March, 2015 to August, 2015 | July, 2019 to February, 2020 |
| Jharkhand | April, 2016 to December, 2016 | January, 2020 to March, 2020 **&** December, 2020 to April, 2021 |
| Odisha | January, 2016 to July, 2016 | January, 2020 to March, 2020 **&** November, 2020 to March, 2021 |
| West Bengal | February, 2015 to July, 2015 | June, 2019 to November, 2019 |
| **North East** |  |  |
| Arunachal Pradesh | April, 2016 to December, 2016 | January, 2020 to March, 2020 **&** December, 2020 to April, 2021 |
| Assam | November, 2015 to March, 2016 | June, 2019 to December, 2019 |
| Manipur | February, 2015 to December, 2015 | July, 2019 to January, 2020 |
| Meghalaya | April, 2015 to September, 2015 | July, 2019 to November, 2019 |
| Mizoram | February, 2016 to October, 2016 | July, 2019 to November, 2019 |
| Nagaland | March, 2016 to October, 2016 | July, 2019 to December, 2019 |
| Sikkim | January, 2015 to August, 2015 | August, 2019 to December, 2019 |
| Tripura | February, 2015 to August, 2015 | July, 2019 to November, 2019 |
| **West** |  |  |
| Dadra & Nagar Haveli | April, 2016 to June, 2016 | July, 2019 to November, 2019 * |
| Daman & Diu | April, 2016 to June, 2016 |  |
| Goa | January, 2015 to April, 2015 | August, 2019 to November, 2019 |
| Gujarat | January, 2016 to June, 2016 | June, 2019 to November, 2019 |
| Maharashtra | April, 2015 to September, 2015 | June, 2019 to December, 2019 |
| **South** |  |  |
| Andaman & Nicobar Islands | April, 2015 to July, 2015 | October, 2019 to February, 2020 |
| Andhra Pradesh | May, 2015 to August, 2015 | July, 2019 to November, 2019 |
| Karnataka | February, 2015 to July, 2015 | July, 2019 to December, 2019 |
| Kerala | March, 2016 to October, 2016 | July, 2019 to December, 2019 |
| Lakshadweep | July, 2016 to September, 2016 | December, 2019 to January, 2020 |
| Puducherry | June, 2015 to July, 2015 | January, 2020 to March, 2020 **&** December, 2020 to March, 2021 |
| Tamil Nadu | February, 2015 to June, 2015 | January, 2020 to March, 2020 **&** December, 2020 to March, 2021 |
| Telangana | February, 2015 to May, 2015 | June, 2019 to November, 2019 |
| **India** | **January, 2015 to December, 2016** | **June, 2019 to April, 2021** |

_Note: *: Dadra & Nagar Haveli and Daman & Diu were combined together._

_NFHS-5 fieldwork for India was conducted in two phases: Phase one from 17th June 2019 to 30th January 2020 and Phase two from 2nd January, 2020 to 30th April, 2021._

**Figure A1: Kernal density curve showing the distribution of haemoglobin level in NFHS4 and NFHS-5**

|  | **NFHS-4** | **NFHS-5** |
| --- | --- | --- |
| **Mean** | 10.546 | 10.224 |
| **SD** | 1.504 | 1.507 |

**Table A2: Age-sex specific prevalence of anaemia among children aged 6-59 months in NFHS-4, & NFHS-5**

| **Age (in months)** | **NFHS-4** | | **NFHS-5** | |
| --- | --- | --- | --- | --- |
|  | **Male** | **Female** | **Male** | **Female** |
| 6-11 months | 69.01 | 67.69 | 78.4 | 76.28 |
| 12-17 months | 72.35 | 69.38 | 81.74 | 78.50 |
| 18-23 months | 70.14 | 69.61 | 79.69 | 79.17 |
| 24-29 months | 65.64 | 63.45 | 74.03 | 73.41 |
| 30-35 months | 59.47 | 59.83 | 69.6 | 70.03 |
| 36-41 months | 54 | 55.58 | 63.94 | 65.06 |
| 42-47 months | 48.01 | 50.54 | 59.88 | 61.05 |
| 48-53 months | 45.43 | 47.47 | 56.22 | 57.44 |
| 54-59 months | 42 | 44.06 | 52.59 | 54.16 |
